# Consumers Respond Positively to the Sensory, Health, and Sustainability Benefits of the Rare Sugar Allulose in Yogurt Formulations

**DOI:** 10.3390/foods11223718

**Published:** 2022-11-19

**Authors:** Margaux R. Mora, Zhixin Wang, Julie M. Goddard, Robin Dando

**Affiliations:** Department of Food Science, Cornell University, Ithaca, NY 14853, USA

**Keywords:** allulose, natural sweeteners, dairy, sustainability, rare sugars, yogurt, sensory

## Abstract

Increased added sugar consumption is associated with type II diabetes, metabolic syndrome, and cardiovascular disease. Low and no-calorie alternative sweeteners have long been used as an aid in the reduction of added sugar. Unfortunately, these alternative sweeteners often have notable sensory deficits when compared to sucrose. Furthermore, many alternative sweeteners have synthetic origins, while consumers are increasingly turning to foods from natural origins, and from more sustainable sources. Such sweeteners include the rare sugar allulose, which can be manufactured from common agricultural waste and dairy co-product streams, and is reported to have a sensory profile similar to sucrose. This study aimed to determine the influence of the rare sugar allulose on consumer perception of sweetened vanilla yogurt. Participants were recruited to evaluate 4 vanilla yogurts sweetened with either sucrose, allulose, stevia or sucralose, and to rate their liking of the samples overall, and for flavor, texture, and their purchase intent. Statistical analysis of hedonic data from 100 consumers suggested that allulose performed similarly to sucrose in liking and purchase intent, and superior to other sweeteners tested in this study, with fewer off-flavors. Moreover, when consumers were queried on their purchase intent after learning details on the sweetener for each formulation, allulose scored significantly higher than all other formulations in purchase intent. This study highlights the potential of the rare sugar allulose as a low calorie, zero glycemic index, natural and better tasting sugar replacement in sweetened yogurt.

## 1. Introduction

Yogurt consumption has steadily increased over recent years due to awareness around the health benefits of fermented foods, and positive associations of their effects on the gut microbiome [1,2]. Sucrose is commonly added to yogurt to improve flavor and reduce any perceived negative sensory attributes (e.g., sourness, bitterness). However, its addition can in turn counter some of the positive health effects associated with yogurt [3]. Across food products, there has been growing demand for a reduction in added sugar to help combat negative health outcomes associated with excessive sugar consumption [4] and in response to the recent revision of the Nutrition Facts label, which requires the inclusion of added sugars [5]. Low- and no-calorie sweeteners have long been employed to help reduce sugar consumption while maintaining sweetness in products, although these alternative sweeteners often have notable sensory deficits, such as lingering sweetness, and bitter or metallic off tastes, when compared to sucrose [6,7]. Additionally, sweeteners deemed “artificial” or “synthetic” are subject to increasingly negative consumer opinions, with consumers favoring those that can be labeled “natural”, despite these definitions being somewhat arbitrary and poorly defined [8,9]. Thus, consumers are increasingly demanding natural and more sustainably produced products, causing an increase in consumption of natural sweeteners such as stevia, monk fruit, and more recently the rare sugar allulose [10]. Rare sugars are monosaccharides with near-equivalent sweetness intensity and functional properties to sucrose, but often with only a fraction of the caloric density. Research has also shown that rare sugars have fewer adverse health impacts and can even provide beneficial effects on postprandial glucose levels, maintaining blood sugar levels [11,12]. Tagatose and allulose are of particular interest as they have been granted Generally Recognized As Safe (GRAS) status by the U.S. Food and Drugs Administration (FDA, Silver Spring, MD, USA) [5], with specific interest in allulose since being granted exemption from labelling as an “added sugar” [13].

Consumers are increasingly looking for alternative sweeteners such as rare sugars due to health concerns associated with some more established sweeteners, such as sucralose, Ace-K, and saccharin [10], and put increasingly more emphasis on sustainability when making food choices [14,15,16]. Currently, allulose is commonly produced through the bioconversion of corn, yet corn derived sweeteners suffer limitations in both consumer perception and environmental sustainability. One solution to this concern is to use agricultural waste products as the starting material to produce rare sugars. The high lipid, protein, and sugar content in agricultural waste streams presents ideal opportunities for biochemical valorization into value added products. In a study by Mintel, most consumers over the age of 18 were worried about the environmental impact of dairy production [17], indicating a particularly useful upcycling source for allulose that could improve consumer perception of the dairy industry. Utilizing waste products such as whey permeate to produce sweeteners to be recycled into dairy products applies the concept of a circular economy, ensuring that resources are used efficiently throughout their lifespan. The goal of this study was to understand consumer perception of and willingness to pay for dairy products formulated with both common and emerging sweeteners.

Here, we compared sucralose, stevia (specifically Rebaudioside A), and allulose to sucrose in an unsweetened vanilla Greek yogurt base to understand the drivers of consumer preferences. Fermented dairy was selected as a model food system with potential towards the long-range goal of a circular dairy production system, to utilize co-product streams from fermented dairy processes and transform those waste products into dairy-derived non-nutritive sweeteners with beneficial functional, health, and sustainability properties. To address the increasing demand for rare sugars, this sustainable system would enable a circular waste stream from which allulose could be upcycled. Research has recently highlighted the biocatalytic transformation of lactose in whey permeate into glucose and galactose, an essential first step in the pathway to rare sugars [18]. Despite its functional, sensory, nutritional, and regulatory benefits, allulose suffers from low awareness amongst consumers outside of Japan where allulose was first commercialized [19]. The potential of producing allulose from food and agricultural waste stream feedstocks further position it to offer sustainability benefits [6,18]. Existing studies and data compiled by the International Food Information Council have shown that despite consumer interests in environmental sustainability, taste remains the key driver in purchasing [20]. Here, we hypothesized that yogurt sweetened with allulose would have similar consumer appeal to that of sucrose sweetened yogurt, and that purchase intent would increase when consumers were informed that the product was made with sustainably produced sweeteners.

## 2. Materials and Methods

### 2.1. Samples—Ingredients and Preparation

Wegmans’ unsweetened non-fat, plain set Greek yogurt (Wegmans Food Markets, Inc, Rochester, NY, USA) was used as the base for samples in the study. Base yogurt was flavored with 1.3% (wt/wt) vanilla extract (McCormick & Company, Baltimore, MD, USA). Sucrose (6%, wt/wt; Wegmans, Rochester, NY, USA), Allulose (11%, wt/wt; AllSWEET, Anderson Advanced Ingredients, Irvine, CA, USA), Rebaudioside A (0.01%, wt/wt; Ingredion, Bridgewater, NJ, USA), and Sucralose (0.02%, wt/wt; Spectrum Chemical, Gardena, CA, USA) were added to the unsweetened vanilla yogurt. Sweetness equivalence (SE) was matched to 6% (%wt/wt) added sugar (sucrose) in vanilla flavored yogurt and was determined via benchtop testing based on literature values [6]. All samples were stirred manually for at least 10 min using a whisk to ensure the distribution of all added ingredients in the unsweetened set Greek yogurt samples.

### 2.2. Participants

All procedures involving human subjects were reviewed and approved by the Cornell University Institutional Review Board for human subject research. Participants were recruited via a listserv maintained by the Cornell Sensory Evaluation Center, consisting of individuals across the Cornell campus and within the Ithaca community. Participants were recruited as over the age of 18, non-smokers, not pregnant, reported no known food allergies, no reported taste or smell deficiencies. Participants provided informed consent for participation in the study and were compensated for their time.

### 2.3. Sample Evaluation and Questionnaire

100 yogurt consumers (panel demographics in Supplemental Table S1) assessed four yogurt samples containing either sucrose, allulose, stevia, or sucralose, in a sequential monadic, repeated measures counterbalanced design. The average age of consumers was 29, and all consumers self-reported to be familiar with yogurt products. Participants received 1 oz samples of each yogurt presented in 2 oz souffle cups with lids. Samples were rated for overall liking and flavor liking on a 9-point hedonic scale. Aftertaste was assessed using a binary “yes/no” question and open-ended comments were requested from participants to describe with more details what they tasted. Just about right (JAR) [21] scaling was used to assess perceived thickness, sweetness and smoothness of yogurt samples. Following hedonic rating, participants were asked to rate how likely they were to purchase the product they sampled. After this initial rating of purchase intent (5-point scale), they were given information on the nature of the sweetener used in the formulation (Table 1, with purchase intent reassessed to measure how this information influenced their purchase intent. All samples were evaluated in the sensory evaluation center of Cornell University, in a plain booth with controlled air and lighting. Each sample was assigned a randomized 3-digit code and all participants evaluated the samples under white light. Participants were also instructed to rinse their mouth with water in between samples to reduce carry over.

### 2.4. Statistical Analysis

Data were determined to be non-normally distributed, thus a Friedman’s test followed with the Dunn’s multiple comparison test was used in each measure to determine statistical significance (assumed when *p* < 0.05) between measures for each sweetener. The top 2 box method was used to compile data from the top two categories of purchase intent scales (very likely to purchase, likely to purchase) for a more instinctive visualization of consumer purchase intent. All statistical analysis was performed with GraphPad Prism version 8 (Dotmatics, Boston, MA, USA). When present in figures, different letters within a figure indicate significant differences at *p* < 0.05.

Penalty analysis was conducted on participant responses to determine which attributes drove a drop in overall liking. Penalty analysis was performed combining information from JAR scales with overall liking data. Analysis was performed using RedJade Sensory Software (version 4.0, RedJade Sensory Solutions LLC, Martinez, CA, USA).

## 3. Results and Discussion

### 3.1. Allulose Performs on Par in Liking with Sucrose in Sweetening Yogurt

**Figure 1:**
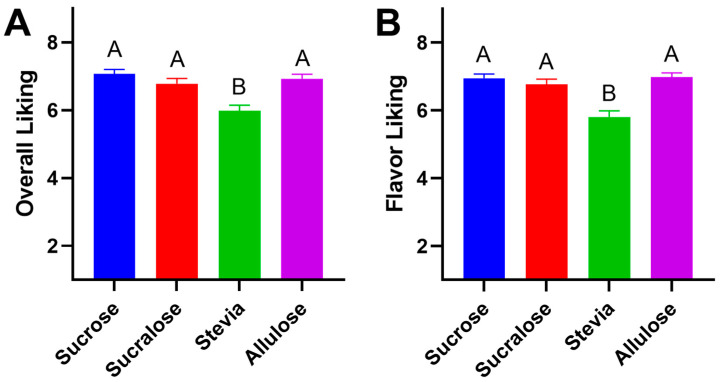
Overall and flavor liking of yogurt samples. 100 participants rated the 4 different yogurt samples on a 9-point hedonic scale for overall liking and flavor liking. The overall liking (**A**) data showed sucrose (7.07 ± 0.14) ranked highest, followed by allulose (6.93 ± 0.14), sucralose (6.78 ± 0.16) and stevia (5.98 ± 0.17). Compiled data for flavor liking (**B**) concluded that allulose (6.98 ± 0.13) ranked highest, followed by sucrose (6.95 ± 0.13), sucralose (6.77 ± 0.16) and stevia (5.81 ± 0.13). Bars show mean and SEM, different letters denote statistical differences.

A notable variation between samples was observed in overall liking. As predicted, stevia was found to be significantly less liked compared to sucrose, allulose, or sucralose (Figure 1). The Friedman’s test followed with the Dunn’s multiple comparison test suggested that the overall liking (Figure 1A) showed no significant difference (*p* > 0.05) when comparing sucrose and sucralose, sucrose and allulose, and sucralose and allulose. A significant difference was found when comparing sucrose and stevia (*p* < 0.001), sucralose and stevia (*p* = 0.006) and stevia and allulose (*p* < 0.001). This difference in overall liking seemed to be driven by flavor, with flavor liking (Figure 1B) also showing no significant difference between allulose, sucralose and sucrose (*p* > 0.05), unlike for stevia. A significantly lower liking was expressed by consumers for stevia versus all other samples (*p* < 0.001). Many studies have noted a high incidence of off flavors in products sweetened with stevia, ranging across food matrices such as tea, yogurt, and chocolate milk [22,23]. Sucralose is often considered to be the most similar low-calorie substitute to sucrose from a sensory perspective, and is the high potency sweetener (HPS) that has the most widespread use in the US market [24]. In a similar vein, allulose has recently gained attention for its similarity to sucrose [25] as well as for additional beneficial functional properties such as potential anti-obesity and antihyperglycemic activity [26,27]. In human subjects, it has been found that allulose has a positive effect in the reduction of fat mass in adults and is effective in suppressing the elevation of blood glucose after food ingestion [12,28,29,30,31,32,33]. In this test, allulose and sucralose were found to be statistically similar from a consumer liking perspective.

### 3.2. Few Sensory Defects Were Reported in Allulose Sweetened Samples

**Figure 2:**
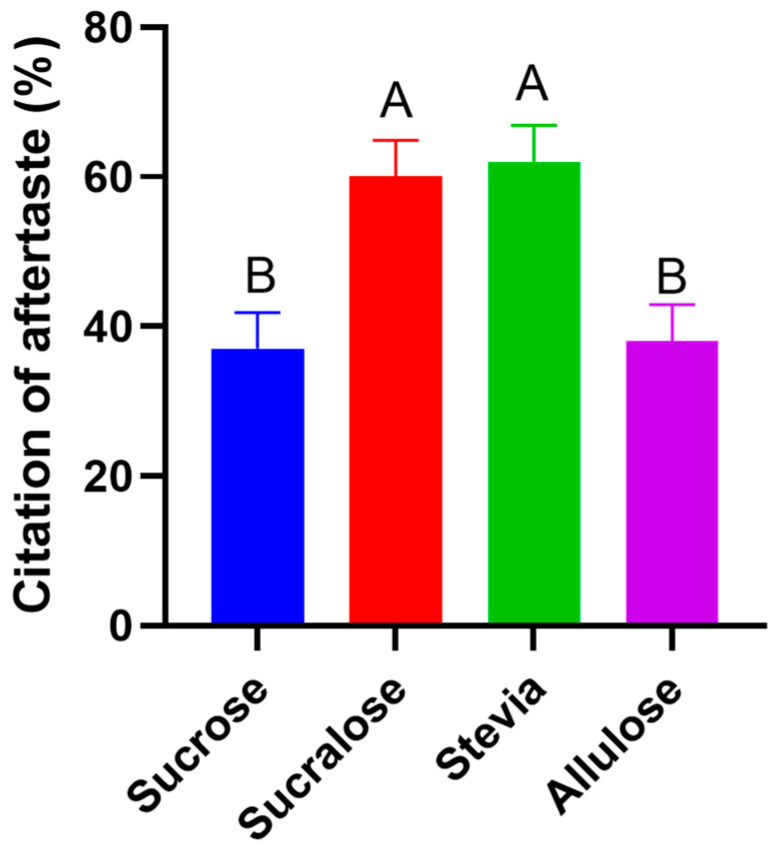
Citation of aftertaste. Proportion of panelists citing an aftertaste in each yogurt sample. Bars show proportion of yes responses, different letters denote statistical differences.

The presence of an aftertaste is often noted in alternative sweeteners and plays a key role in distinguishing their sensory properties from that of sucrose. Alternative sweeteners often have a broad sweetness curve with lingering off-flavors [34]. Here, we found that both sucralose and stevia were cited as having an aftertaste significantly more often than sucrose or allulose (Figure 2). Furthermore, participants noted that the aftertaste associated with sucralose and stevia tasted “artificial”, “chemical-like”, and “bitter” (Supplemental Table S2).

**Figure 3:**
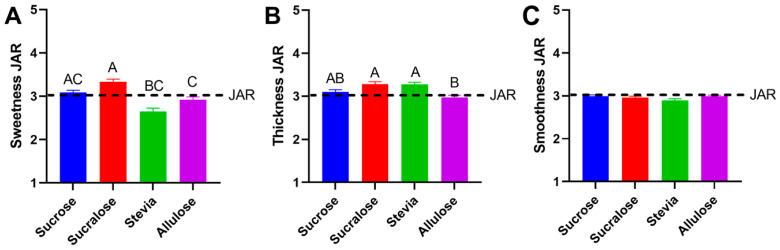
Just-about-right (JAR) ratings of sweetened yogurt samples. JAR data compiled from 100 sensory participants for sweetness (**A**), thickness (**B**), and smoothness (**C**). Friedman’s test followed with Dunn’s multiple comparison test was used in each set to determine statistical significance (assumed when *p* < 0.05) between each pair. JAR rating from 1 to 5 was described as 1 = not sweet enough, 2 = somewhat not sweet enough, 3 = just about the right sweetness, 4 = somewhat too sweet and 5 = much too sweet. Bars show mean and SEM, different letters denote statistical differences.

Yogurt samples were formulated to have equivalent sweetness levels for all sweeteners used. The JAR data for sweetness showed an average rating of 3.09 ± 0.05 for sucrose, 2.92 ± 0.06 for allulose, 2.65 ± 0.08 for stevia and 3.33 ± 0.06 for sucralose. Despite slight differences in scores for sweetness, no significant difference between the samples sweetened with sucrose and allulose was found, whereas a difference was found in the sweetness between sucrose and stevia (*p* = 0.002), sucralose and stevia (*p* < 0.001) and sucralose and allulose (*p* = 0.001) (Figure 3A). No difference in sweetness suggests that a direct comparison can be made between sucrose and allulose as well as between allulose and stevia. Alternatively, slight differences in sweetness may be attributed to the types of sweeteners utilized. Both bulk sweeteners, allulose and sucrose, were similarly rated while the high potency sweeteners (HPS) sucralose and stevia exhibited more variation. Previous studies have demonstrated greater deviation in sweetening power for HPSs [35] across concentrations, which could potentially account for the differences observed here. Furthermore, high notes of bitterness when stevia is used at high levels [6,36] can limit its sweetening capacity due to mixture suppression [22,37,38]. In fact, in examining open-ended comments, the word “bitter” was used by 7 participants when testing the yogurt sweetened with allulose, compared to more than double (15) using the same descriptor for the sample sweetened with stevia (Supplemental Table S2).

Samples were also rated on JAR scales for thickness and smoothness, as sweeteners like sucrose are known to act as a bulking agent, and thus add more than just sweetness to food products. The JAR data for the thickness showed an average rating of 3.10 ± 0.05 for sucrose, 2.97 ± 0.05 for allulose, 3.27 ± 0.06 for stevia and 3.28 ± 0.06 for sucralose. Interestingly, the allulose sweetened sample was rated as slightly less thick versus stevia or sucralose sweetened samples, despite presumably benefiting from the bulking properties inherently missing from the HPSs. No difference was reported in smoothness between samples (Figure 3B,C), with all samples rated close to JAR.

### 3.3. Consumer Perception’s Impact on Purchase Intent

**Figure 4:**
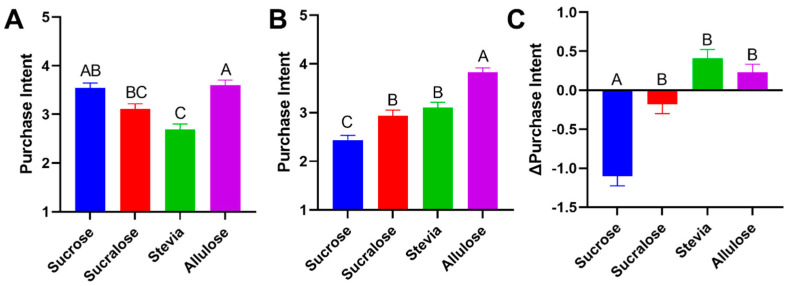
Purchase intent. Purchase intent (5-point scale) for the samples was compiled with data corresponding to before (**A**) and after (**B**) providing information about the sweeteners to participants, as well as the difference between before and after, presumably arising from this information (**C**). In the scoring process, 1 = Definitely would not purchase, 2 = Probably would not purchase, 3 = May or may not purchase, 4 = Probably would purchase, 5 = Definitely would purchase. Bars show mean and SEM, different letters denote statistical differences.

Although taste remains the greatest driver of purchase intent, consumers have additional considerations when choosing between products such as healthfulness, sustainability, convenience, and price [20]. To determine the influence of information about the source of the sweetener, the amount of added sugar, and the potential benefit towards sustainability, consumers were provided with additional informational statements about each sweetener and queried a second time about purchase intent (Table 1, Figure 4).

**Figure 5:**
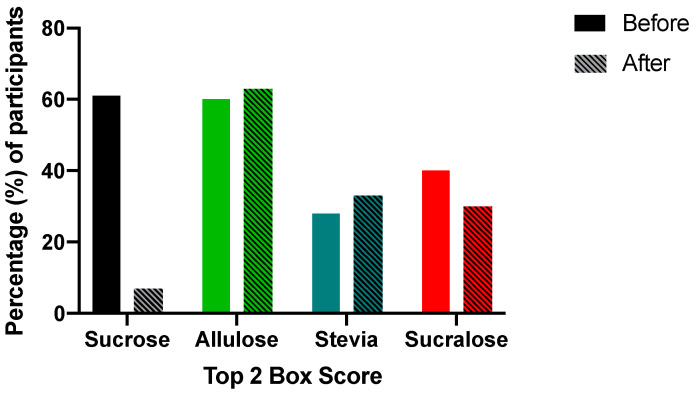
Top 2 box score purchase intent. The top 2 box score showed that natural sweeteners (allulose and stevia) were more likely to be purchased when information was disclosed, whereas sucrose and sucralose’s purchase intent decreased when information about those sweeteners was given to the consumers. Bars indicate percent of panelists who probably of definitely would purchase on sensory properties alone, with hatched bars after informational statement.

In initial scoring of purchase intent, all sweeteners aside from stevia performed favorably, in a manner which aligned well with scores of overall liking (Figure 4A), with top-2-box scores for sucrose, sucralose and allulose-sweetened yogurt at 61, 40 and 60% of panelists, respectively, compared to stevia at 28 (Figure 5). Performing statistical analysis on the data suggested no significant difference (*p* > 0.05) in pre-informed purchase intent between sucrose and sucralose, sucrose and allulose and sucralose and stevia. A significance difference in pre-informed purchase intent was found when comparing sucrose and stevia (*p* < 0.001), sucralose and allulose (*p* = 0.04) and stevia and allulose (*p* < 0.001). When compiling the informed purchase intent data, trends changed dramatically. Sucralose and stevia showed no significant difference between purchase intent, however after informational statements (Table 1) designed to be similar to those that may be given on packaging or on food labeling (ingredients, calories, claims), allulose and stevia-sweetened yogurts purchase intent scores improved (Figure 4B), whereas both sucrose and sucralose-sweetened yogurts declined (Supplemental Table S3). This change in purchase intent demonstrates clearly that consumers do not only take sensory features into account when determining the products they are likely to purchase, that consumers are seeking to reduce their consumption of sucrose, and that consumers value natural sweeteners. The shift in purchase intent is clearly demonstrated in top 2 box scores, that show how the proportion of consumers likely to purchase each sample altered in response to consumers viewing statements related to the products they tasted (Figure 5). Statements were centered around either healthfulness (using the key term “added sugar”), sustainability (using the key terms “upcycled”), and natural/artificial labeling (which were explicitly stated). Although allulose only experienced a 3% increase in purchase intent when presented with the informational statement, its original score was high (Figure 5), and resultantly was higher than any other sample after information was provided (Figure 4B) with the highest top-2-box scores in informed condition by 30%. On the other hand, sucrose suffered a drop in purchase intent once consumers were presented with the statement on added sugar content, even though it was explicitly stated this level was “typical for this product”.

Results were in line with a greater consumer focus on healthfulness and the emergence of many new diets and lifestyles, such as keto and low carb. While stevia did see an increase in purchase intent when information was provided, this increase was not sufficient to distinguish its scores from those of sucralose, confirming that a natural claim may not be effective alone in driving purchase intent [39], and is best when combined with a pleasing sensory profile. Comparing purchase intent before and after disclosure of the potential source of the sweetener confirmed that consumers were willing to purchase products made with upcycled ingredients when the flavor of the product was acceptable while helping to improve the sustainability of our food system [40].

**Figure 6:**
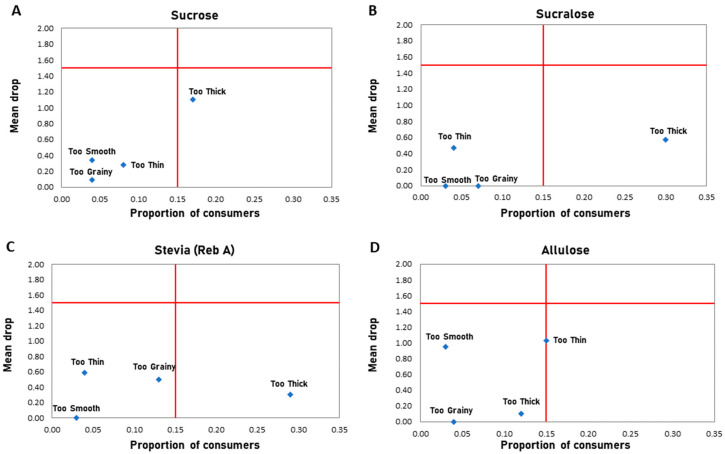
Penalty analysis of sweetened yogurt samples. Penalty analysis showing the attributes negatively impacting the overall liking (mean drop), and proportion of consumers agreeing for yogurt products sweetened with (**A**) Sucrose, (**B**) Sucralose, (**C**) Stevia, (**D**) Allulose.

Penalty analysis was conducted to determine which attributes drove the liking or disliking of each sweetened yogurt sample (Figure 6). Typically, formulations with high potency sweeteners can be considered lacking in mouthfeel and are often perceived as being too thin [41], however, here we saw that sucralose and stevia were considered to be “too thick” (Figure 6B,C). While sucrose and allulose had higher mean drops due to a single textural attribute (Figure 6A,D), sucralose and stevia experienced a mean drop from a greater proportion of consumers (Figure 6B,C). This drop by a greater proportion of consumers suggests that high potency sweeteners exhibit a clear difference in texture perception compared to bulk sweeteners and outlines a critical area for improvement for products that rely on alternative sweeteners.

#### Practical Implications and Future Directions

Challenges regarding the use of rare sugars in food products include their high cost and uncertainty about consumer acceptance towards this novel ingredient. Research on developing technologies to valorize agricultural waste materials into rare sugars offers an approach to reduce these costs. This study quantified consumer perception and acceptance of allulose in dairy products such as yogurt. Our data indicate a positive consumer reception of allulose, a less known natural sweetener in the United States, in yogurt. Greek yogurt was used as a model system in this study to evaluate different sweeteners, but the information collected may also be helpful in other product sectors. Although our study focused on liking of allulose using yogurt as a model medium, our data are only sensory in nature, and additional research such as a cost–benefit analysis would further improve understanding of the use of allulose in specific applications.

## 4. Conclusions

We conclude that allulose, especially when produced in a sustainable fashion, is a promising sweetener that can appeal to a growing segment of environmentally informed consumers interested in reducing their added sugar consumption. Allulose-sweetened yogurt displayed high purchase intent before the disclosure of sustainability information, presumably rooted in its pleasing sensory profile, which further increased on panelists’ learning of its content, suggesting consumers do not view allulose negatively, as they seemed to with sucralose. Sucralose exhibited a decrease in purchase intent when labeled as an artificial sweetener, although its overall liking was relatively high as expected, and in line with its well-reported pleasing sensory properties. Although it maintains high performance in the market, consumer distaste for artificially labeled foods may in the future turn them away from purchasing products containing sucralose. While purchase intent for stevia improved alongside natural labeling claims, it still suffered from low consumer liking, in line with previous reports of poor sensory performance. Finally, purchase intent for sucrose dropped a marked 54% after consumers were given information including the percentage of added sugar, illustrating the consumer shift away from foods high in added sugars, despite enjoying their taste. With this consumer focus on natural and more healthful foods, future work should focus on improving consumer recognition of allulose as well as the characterization of additional natural sweeteners.

## Figures and Tables

**Table 1:** Statements influencing purchase intent.

Sweetener	Information Provided
Sucrose	The sample that you have just tasted contains 10 g of added sugar per serving (20% of your daily recommended intake of added sugar), which is typical for this product.
Allulose	The sample that you have just tasted contains 0 g of added sugar and gets its sweetness from allulose, an upcycled natural sweetener that is produced in a sustainable way.
Stevia (Reb A)	The sample that you have just tasted contains 0 g of added sugar and gets its sweetness from stevia, a natural sweetener.
Sucralose	The sample that you have just tasted contains 0 g of added sugar and gets its sweetness from sucralose, an artificial sweetener.

## Data Availability

Data available from authors on request.

## Supplementary Materials

The following supporting information can be downloaded at: https://www.mdpi.com/article/10.3390/foods11223718/s1, Table S1. Panel demographics, Table S2. Changes in purchase intent, Table S3. Open-ended comments of attributes.
