# Supplementary material for: Consumers Respond Positively to the Sensory, Health, and Sustainability Benefits of the Rare Sugar Allulose in Yogurt Formulations
Source: Foods. 2022 Nov 19;11(22):3718. doi: 10.3390/foods11223718 (PMC9689152; doi:10.3390/foods11223718)
Supplement: Supplementary file 1 [file foods-11-03718-s001.zip › foods-2027477-supplementary.pdf]

Supplementary Materials

Table S1. Panel demographics.

Gender	Age (Years)	Dietary Preferences	Frequency of Consumption of Alternative Sweeteners	Preference for Natural Foods
69% female	29.2 ± 9.5	5%, clean label	9%, daily	26%, care that the food they consume is natural
31% male		6%, sustainable	11%, more than 3 times a week	31%, care that foods do not contain artificial ingredients
		17%, high protein	16%, 2-3 times a week	33%, do not care about artificial ingredients and/or are indifferent to labeling
			16%, once a week	
			17%, once a month	
			31%, never	

Gender, dietary preferences, consumption frequency, and natural foods preference are expressed as percent. Age is expressed as the mean in number of years old with standard deviation.

Table S2. Open-ended comments of attributes.

Sweetener	Bitterness	Artificial Taste	Astringent/Drying	Sour/Tangy	Vanilla
Sucrose	6	3	2	9	4
Allulose	7	0	6	19	8
Sucralose	10	8	8	10	11
Stevia (Reb A)	15	15	6	28	1

Table S3. Changes in purchase intent

	Sucrose		Allulose		Stevia		Sucralose	
Score	Before	After	Before	After	Before	After	Before	After
1	2	19	5	1	17	10	5	16
2	19	27	8	4	28	16	28	18
3	18	47	27	32	27	41	27	36
4	45	5	42	37	25	20	31	17
5	16	2	18	26	3	13	9	13

Purchase intent of the 100 participants was first compiled based on initial sensory preferences of the samples without disclosure of the sweetener used. Following a purchase intent preference, the participants were given information about the specific sweetener, how it would show on a nutritional label, whether it is natural or not as well as its potential source. Participants were asked to score their purchase intent again after receiving that information. In the scoring process, 1=Definitely would not purchase, 2=Probably would not purchase, 3=May or may not purchase, 4=Probably would purchase, 5=Definitely would purchase.
